# Pigeonpea genotypes influence parasitization preference and survival and development of the *Helicoverpa armigera* larval parasitoid, *Campoletis chlorideae*

**DOI:** 10.1186/2193-1801-3-378

**Published:** 2014-07-28

**Authors:** Shiddalingappa V Hugar, Hari C Sharma, Kondikallu Basavan Goud

**Affiliations:** International Crops Research Institute for the Semi-Arid Tropics, Patancheru, 502324 Andhra Pradesh India; Department of Agricultural Entomology, College of Agriculture, University of Agricultural Sciences (UAS), Dharwad, 580005 Karnataka, India

**Keywords:** Pigeonpea, *Helicoverpa armigera*, *Campoletis chlorideae*, Biological control, Plant resistance, Tritrophic interactions, Compatibility

## Abstract

Studies were undertaken to identify pigeonpea, *Cajanus cajan* (L.) Millspaugh and the wild relative of pigeonpea, *Cajanus scarabaeoides* (L.) (accession ICPW 125,) genotypes that are hospitable to the pod borer, *Helicoverpa armigera* (Hübner) (Lepidoptera: Noctuidae) larval parasitoid, *Campoletis chlorideae* Uchida (Hymenoptera: Ichneumonidae) for the management of this pest in pigeonpea based cropping systems. Percentage parasitization of the *H. armigera* larvae by the *C. chlorideae* females was greater under no-choice conditions than under multi-choice conditions because of forced parasitization under no-choice conditions. Lowest parasitization was recorded on the wild relative, ICPW 125, which may be due to long nonglandular hairs and low survival of *H. armigera* larvae. Parasitization of *H. armigera* larvae was greater under no-choice, dual-choice and/or multi-choice conditions on ICPL 87, ICPL 87119 and ICPL 87091, which are susceptible to *H. armigera*, than on the pod borer-resistant genotypes ICPL 332WR, ICPL 84060 and ICPB 2042; while survival and development of the parasitoid was better on *H. armigera* larvae fed on ICPL 87, ICPL 87119, LRG 41, ICP 7035 and ICPL 87091 than on ICPL 332WR, ICPL 84060, ICPB 2042 and ICPW 125. The genotypes ICPL 87, ICPL 87119, LRG 42 and ICPL 87091 that are hospitable to *C. chloridae*, are better suited for use in integrated pest management to minimize the losses due to *H. armigera* in pigeonpea.

## Introduction

Pigeonpea is one of the major pulses grown in the semi-arid tropics between 30°N and 30°S, covering about 50 countries in Asia, Africa and the Americas. It is damaged by more than 200 species of insects, of which *Helicoverpa armigera* (Hubner) (Lepidoptera: Noctuidae) is the most important pest in the semi-arid tropics (Reed et al. 1989; Sharma 2005). *Helicoverpa armigera* has a wide host range, and feeds on more than 250 crop species. It has developed very high levels of resistance to conventional insecticides, including synthetic pyrethroids (Kranthi et al. 2002). *Helicoverpa armigera* is a polyphagous pest (Bilapate 1984; Firempong and Twine 1986), damaging a wide range of agricultural crops including cotton, tomato, sunflower, grain legumes, vegetables, cereals and fruit crops. It causes an estimated annual loss of over $350 million in pigeonpea, and over $2 billion in the semi-arid tropics on different crops despite application of insecticides costing over $500 million annually (Sharma 2005).

Evaluation of more than 14,000 pigeonpea accessions for resistance to *H. armigera* has revealed low to moderate levels of resistance to this pest (Reed and Lateef 1990; Lateef and Pimbert 1990), and several genotypes with moderate levels of resistance to *H. armigera* have been identified (Patnaik et al. 1989; Lateef and Pimbert 1990; Borad et al. 1991; Kalariya et al. 1998). Varieties with moderate levels of resistance to pod borers have also been developed, and/or released for cultivation to the farmers (Sharma et al. 2005, 2010). Genotypes such as ICPL 187-1, ICP 7203-1, ICPL 98008, T 21, ICP 7035 and ICPL 332 have shown moderate levels of resistance to *H. armigera* across planting dates (Kumari et al. 2010a). The *H. armigera* larvae reared on leaves or pods of ICPL 332, ICPL 84060, ICP 7035, ICPL 88039 and T 21 exhibit reduced larval and pupal weights, and prolongation of larval and pupal developmental periods, suggesting that antibiosis is one of the mechanisms of resistance, which may have implications for survival and development of the natural enemies of this pest (Kumari et al. 2010b).

Over 250 natural enemies have been recorded on *H. armigera* (Romeis and Shanower 1996), of which, the egg parasitoids, *Trichogramma* spp. and the larval parasitoids, *Campoletis chlorideae* Uchida (Hymenoptera: Ichneumonidae), *Carcelia illota* Curran, *Palexotista* spp., and *Goniozu*s spp. are predominant parasitoids of *H. armigera* in different agro-ecosystems. However, the activity and abundance of natural enemies varies across crops (Pawar et al. 1986), and different genotypes of the same crop (Romeis and Shanower 1996; Sharma et al. 2003; Dhillon and Sharma 2007). Host plant selection by the female parasitoids, involves a series of complex responses in a non-random manner to a hierarchy of physical and/or chemical stimuli that lead them to their potential hosts (Vet and Groenewold 1990; Lewis et al. 1991; Tumlinson et al. 1993). Parasitoids also respond to the volatiles emanating from both undamaged (McAuslane et al. 1990; Li et al. 1992; Turlings and Tumlinson 1992; Udayagiri and Jones 1992) and damaged (Whitman 1988; Turlings et al. 1990, 1995; Mattiaci et al. 1994; de Moraes et al. 1998; War et al. 2011) plants.

Genotypic resistance has a considerable influence on parasitism of insect pests in different crops. The nature of influence depends on the insect pest, natural enemy, and the crop (Sharma et al. 2003). The rates of parasitism by *Campoletis sonorensis* (Cameron) and *Cotesia congregata* (Say) have been reported to be significantly lower on the resistant wild tomato, *Lycopersicon hirsutum f. glabratum* (accession PI 134417), but had little effect on parasitism by *Cotesia marginiventris* (Cresson) and *Cardiochiles nigriceps* Viereck (Farrar et al. 1994). In chickpea, parasitism of *H. armigera* larvae by *C. chlorideae* ranged from 8.33 to 28.00% (Gupta and Raj 2003), and varied considerably across genotypes (Kaur et al. ( 2004). However, there is no information on genotypic effects on the activity and abundance of natural enemies in pigeonpea. The present studies were undertaken to study the effect of different genotypes of pigeonpea on the parasitization of *H. armigera* larvae by *C. chlorideae* to identify genotypes that are compatible with the natural enemies of this pest.

## Material and methods

### Experimental material

#### Plant material

Eight pigeonpea genotypes (ICPL 87, ICPL 87119, and ICPL 87091–susceptible, LRG 41, T 21, and ICP 7035–moderately resistant, and ICPL 84060, ICPL 332 WR and ICPB 2042 (Hairy pods) - resistant) and one accession of wild relative of pigeonpea, *Cajanus scarabaeoides* (L.) (ICPW 125) were grown in the field during the rainy season (June-Dec) in deep black soils (Vertisols). The test genotypes were planted twice at 30 day intervals to produce flowers and pods of all genotypes at the same time. Normal agronomic practices, as per ICRISAT procedure, were followed for raising the crop, but without insecticide application. The inflorescences were covered with a nylon bag at the initiation of flowering (flower bud formation) to protect the test samples from natural infestation by the insects.

### Maintenance of insect cultures

#### Pod borer, *H. armigera*

The *H. armigera* culture was initiated by collecting the larvae from the farmers’ fields and reared on the natural host for one generation before being introduced into the laboratory culture to avoid contamination with the nuclear polyhedrosis virus, bacteria or fungi. The *H. armigera* was reared on a chickpea flour based semi-synthetic artificial diet (Armes et al. 1992).

#### Larval parasitoid, *C. chlorideae*

The cocoons of *C. chlorideae* were collected from chickpea fields and kept individually in a glass vial (2 cm in diameter, and 10 cm in length) and plugged with cotton wool. Twenty newly eclosed female adult wasps were kept in each cage (10 cm diameter × 20 cm length, and covered with a lid having 60 wire-mesh) with other virgin wasps. A cotton swab with 10% sucrose solution was placed inside the cage as a food for the parasitoid adults. After mating, the females were used for parasitization of second instar *H. armigera* larvae, and then reared on chickpea based artificial diet as described by Sharma et al. ( 2008). The culture was maintained at 27 ± 2°C, 65–75% RH, and 12 h photoperiod. Mated females and progeny of the same parent (originating from isofemales, i.e. culture initiated from a single female) were used for the experiments.

### Effectiveness of *C. chlorideae*in parasitization of *H. armigera*larvae on different genotypes of pigeonpea

The effect of different genotypes of pigeonpea on parasitization of *H. armigera* larvae by the females of *C. chorideae* was studied under no-choice, dual-choice, and multi-choice conditions. For this purpose, the inflorescences (30 cm long) of different genotypes at the initiation of podding (with 50% flowers) were cut with a sharp knife, and immediately placed in a 100 ml conical flask containing 75 ml of one per cent sucrose solution, and held in an upright position using cotton wool. The inflorescences were infested with the 30 laboratory-reared second-instar *H. armigera* larvae. After 6 h, the infested inflorescences were exposed to the three, six and ten pairs of *C. chlorideae* females under no-choice, double-choice and multi-choice conditions, respectively inside a cage (30 × 30 × 30 cm). The wasps were provided with 10% sucrose solution on a cotton swab as a food. After 48 h, the larvae were collected from the infested inflorescences and placed individually in 15 ml glass vials containing 10 g artificial diet. The larvae were reared on the artificial diet until cocoon formation.

#### No-choice conditions

Under no-choice conditions, a single inflorescence of the test genotype was kept inside a wooden cage (30 × 30 × 30 cm). Each experiment was repeated twice, and each set replicated 10 times.

#### Dual-choice conditions

Under dual-choice conditions, the *C. chlorideae* females were offered the choice between the infested inflorescence of the test genotype and of the susceptible check, ICPL 87 placed at the opposite ends of a wooden cage. (30 × 30 × 30 cm). Each experiment was repeated 10 times. After 48 h, the relative parasitization (%) preference was computed as follows.


No effect on parasitization = No difference in relative parasitization between the test genotype and the susceptible check, ICPL 87.

Negative effect on parasitization = Parasitization of the *H. armigera* larvae on the test genotype is significantly lower than on the susceptible check, ICPL 87.

Positive effect on parasitization = Parasitization of *H. armigera* larvae on the test genotype is significantly greater than on the susceptible check, ICPL 87.

#### Multi-choice conditions

Under multi-choice conditions, the inflorescences of all the 10 test genotypes were arranged in a circular arena inside a wooden cage (60 × 60 × 60 cm). The experiment was conducted in a completely randomized block design, with two sets having three replications in each set. Observations were recorded on the numbers of *H. armigera* larvae recovered, and the proportion of larvae parasitized by *C. chlorideae*. For recording the numbers of *H. armigera* larvae parasitized by *C. chlorideae*, the larvae collected from inflorescences of one set were dissected immediately by placing them in a drop of Ringer’s solution on a glass slide under a stereomicroscope, and the percentage parasitization was calculated as follows (Tian et al. 2008).


The second set was used to record the survival and development of *C. chlorideae* as follows;


Data were also recorded on egg and larval period, pupal period, and sex ratio of the parasitoid.

### Statistical analysis

The data from no-choice, dual-choice and multiple choice conditions were subjected to analysis of variance using Genstat 13th edition. Significance of differences between the genotypes under no-choice and multi-choice conditions was tested by F-test, while the significance of differences between the genotypic means was judged by least significant difference at P = 0.05. Under dual-choice conditions, the significance of differences between the test genotypes and the susceptible check was judged by the students’ paired t-test at P = 0.05.

## Results

### Effectiveness of *C. chlorideae*in parasitization of *H. armigera*larvae on different genotypes of pigonpea

#### No-choice conditions

**Figure 1:**
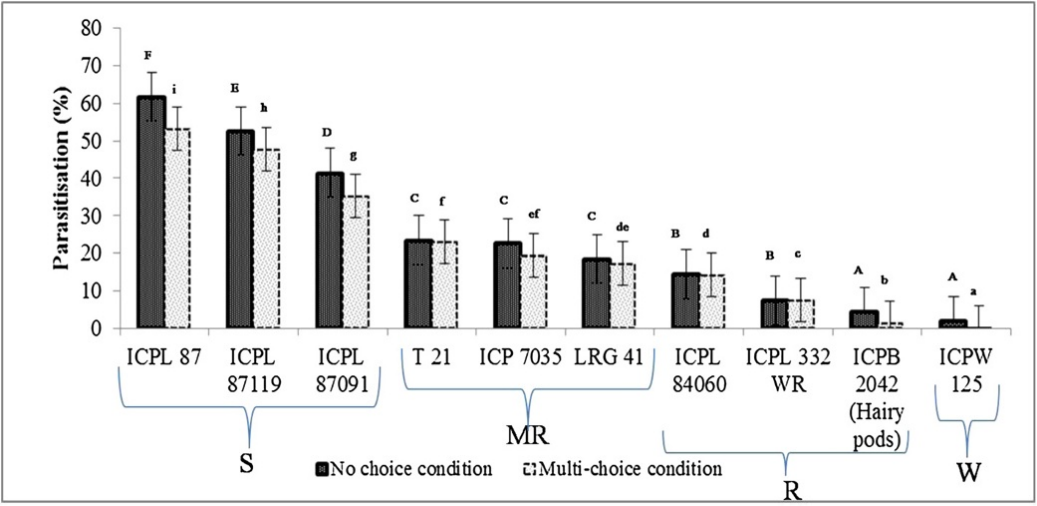
**Influence of different pigeonpea genotypes on parasitization of** ***H. armigera*** **larvae by** ***C. chlorideae*** **females under no-choice and multi-choice conditions.** The bars followed by the same letter (upper or lower case) are not statistically significant at P ≤ 0.05. S-Susceptible, MR-Moderately resistant, R-Resistant, and W-Wild resistant.

Under no-choice cage conditions, there were significant differences in larval parasitization on different pigeonpea genotypes, and highest parasitization was recorded on ICPL 87 (61.74%), followed by ICPL 87119 (52.55%), ICPL 87091 and (41.39%) (Figure 1). Larval parasitization was quite low (4.50–23.41%) on T 21, ICP 7035, LRG 41, ICPL 84060, ICPL 332WR, and ICPB 2042, suggesting that these genotypes were not hospitable to the larval parasitoid, *C. chlorideae*. Least larval parasitization was observed on the pigeonpea wild relative, *C. scarabaeoides* accession ICPW 125 (2.00%), probably because of the presence of long hairs and/or chemicals that may be repellent to the adult wasps, and the possible adverse effects of secondary metabolites in this accession on the parasitoid survival and development.

#### Dual-choice conditions

**Figure 2:**
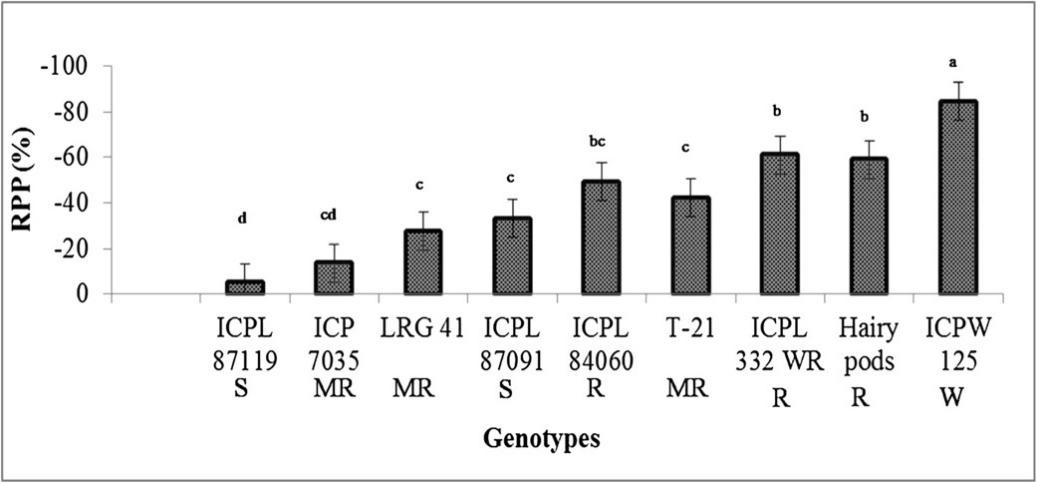
**Parasitization of 2** ^**nd**^ **instar larvae of** ***H. armigera*** **by** ***C. chlorideae*** **females on different genotypes of pigeonpea under dual-choice conditions.** The bars followed by the same letter are not statistically significant at P ≤ 0.05. RPP = Relative parasitization preference in relation to the susceptible check, ICPL 87. S-Susceptible, MR-Moderately resistant, R-Resistant, and W-Wild resistant.

**Figure 3:**
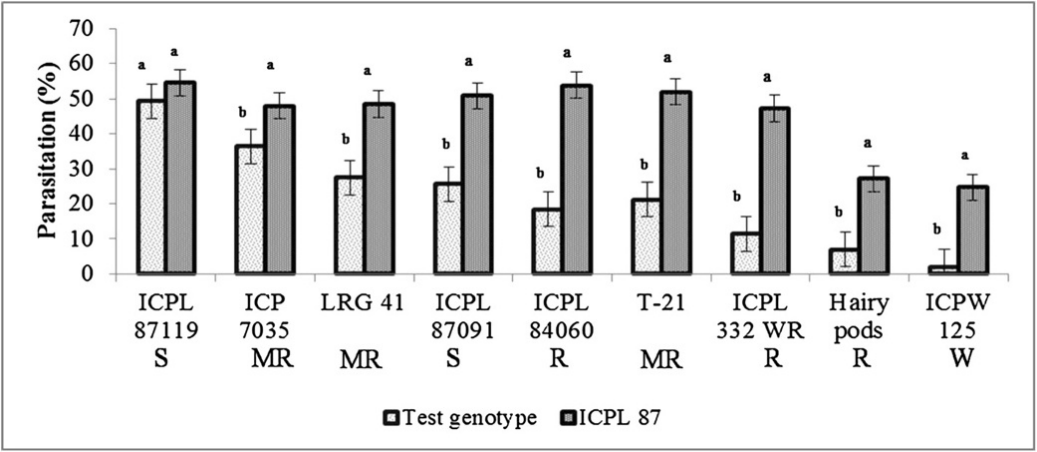
**Parasitization preference of** ***C. chlorideae*** **females towards 2** ^**nd**^ **instar larvae of** ***H. armigera*** **on different genotypes of pigeonpea in relation to the susceptible check, ICPL 87 under dual-choice conditions.** A pair of bars followed by the same letter are not statistically significant at P ≤ 0.05. S-Susceptible, MR-Moderately resistant, R-Resistant, and W-Wild resistant.

Under dual-choice conditions, when the parasitoid females were offered a choice between the test cultivar and the susceptible check, ICPL 87, greater parasitization was recorded in the larvae released on ICPL 87 than on the test cultivars. The differences were much larger in case of ICPL 84060 (18.39 vs 53.82%), T 21 (21.14 *vs* 51.94%), ICPL 332 WR (11.43 vs 47.20%) and ICPW 125 (2.08vs 24.62%) (Figure 2). The relative parasitization preference in relation to the susceptible check, ICPL 87 (RPP) by *C. chlorideae* on different test genotypes as compared to ICPL 87 ranged from -5.01 to -84.2%. Highest RPP of -84.2% was observed on ICPW 125, followed by ICPL 332WR (-61.01%), ICPB 2042 (-58.96%), ICPL 84060 (-49.07%) and T 21 (-42.14%) (Figure 3).

#### Multi-choice conditions

Under multi-choice conditions, percentage larval parasitization was highest on the susceptible check, ICPL 87 (53.33%), followed by ICPL 87119 (47.67%). Least parasitization was recorded on *C. scarabaeoides* accession, ICPW 125 (0.13%), followed by ICPB 2042 (1.32%). The percentage parasitization of *H. armigera* larvae on rest of the genotypes varied from 7.46 to 35.33% (Figure 1).

### Effect of different pigeonpea genotypes on the development and survival of *C. chlorideae*

Egg + larval period of *C. chlorideae* reared on *H. armigera* larvae fed on the susceptible check, ICPL 87 was shortest (7.33 days), followed by those fed on ICPL 87119 (8.33 days). The egg + larval developmental period of *C. chlorideae* was prolonged when the *H. armigera* larvae were reared on ICP 7035 (11.00 days), ICPB 2042 (12.00 days), ICPL 332 WR (12.33 days) and ICPW 125 (16.00 days) (Table 1). The pupal period of *C. chlorideae* was shorter when the *H. armigera* larvae were fed on ICPL 87119 (7.68 days), followed T 21 (8.07 days), ICPL 87 (8.21 days) and ICPL 87091 (8.64 days) as compared to those fed on ICPL 332WR (9.40 days), ICP 7035 (9.57 days), ICPL 84060 (9.83 days) and LRG 41 (9.87 days). Pupal period of *C. chlorideae* was prolonged by 4–6 days when the *H. armigera* larvae were reared on the flowers/pods of ICPB 2042 and ICPW 125 (Table 1).

**Table 1:** **Development and survival of** ***Campoletis chlorideae*** **parasitizing 2** ^**nd**^ **instar** ***H. armigera*** **larvae on different genotypes of pigeonpea (ICRISAT, Patancheru, 2009-2010)**

Genotypes	Egg + larval period (days)	Pupal period (days)	Cocoon formation (%)	Adult emergence (%)	Adult longevity (days)
ICPL 87 **S**	7.33 ± 0.33^a^	8.21 ± 0.15^ab^	22.03 ± 0.60 (27.98 ± 0.62)^b^	17.00 ± 1.15 (24.32 ± 1.17)^d^	23.00 ± 3.06^c^
ICP 7035 **MR**	11.00 ± 0.58^d^	9.57 ± 0.43^c^	33.57 ± 1.83 (35.39 ± 1.94)^a^	40.33 ± 4.06 (39.38 ± 4.43)^a^	29.00 ± 1.15^d^
LRG 41 **MR**	9.33 ± 0.34^bc^	9.87 ± 0.13^c^	19.91 ± 1.42 (26.47 ± 1.45)^b^	39.67 ± 0.87 (39.03 ± 0.96)^a^	20.00 ± 3.46^bc^
ICPL 87091 **S**	9.33 ± 0.32^bc^	8.64 ± 0.18^abc^	20.25 ± 0.64 (26.74 ± 0.65)^b^	24.00 ± 2.08 (29.29 ± 2.15)^c^	29.70 ± 0.88^d^
ICPL 84060 **R**	9.00 ± 0.10^bc^	9.83 ± 0.17^c^	19.93 ± 0.93 (26.50 ± 0.95)^b^	33.00 ± 0.56 (35.05 ± 0.61)^b^	18.70 ± 0.33^bc^
T 21 **MR**	9.73 ± 0.18^c^	8.07 ± 0.30^a^	20.14 ± 0.71 (26.67 ± 0.72)^b^	15.67 ± 1.20 (23.28 ± 1.22)^d^	19.00 ± 0.58^bc^
ICPL 332 WR **R**	12.33 ± 0.88^e^	9.4 ± 0.87^bc^	14.28 ± 0.62 (22.19 ± 0.63)^c^	9.67 ± 1.10 (18.05 ± 1.20)^e^	18.00 ± 0.58^bc^
ICPB 2042 (Hairy pods) **R**	12.00 ± 0.57^de^	12.17 ± 0.17^d^	14.70 ± 1.32 (22.50 ± 1.33)^c^	5.33 ± 0.86 (13.26 ± 0.87)^f^	14.33 ± 1.76^ab^
ICPW 125 **W**	16.00 ± 0.56^f^	14.48 ± 0.37^e^	9.00 ± 0.58 (17.44 ± 0.57)^d^	3.33 ± 0.87 (10.34 ± 0.86)^f^	10.70 ± 0.88^a^
SE±	0.425	0.404	0.74	1.20	1.718
LSD (P _0.05_)	1.264^*^	1.202^*^	2.98^*^	4.83^*^	5.11^*^

Values in parentheses are Arcsine transformed. The values followed by the same letter within a column are statistically non-significant at P ≤ 0.05.

S-Susceptible, MR-Moderately resistant, R-Resistant, and W-Wild resistant.

Percentage cocoon formation of *C. chlorideae* was greater when the *H. armigera* larvae were fed on ICP 7035 (33.57%), followed by the larvae fed on ICPL 87119 (31.81%). Cocoon formation ranged from 19.91–22.03% when the *H. armigera* larvae were reared on ICPL 87, T 21, ICPL 84060, ICPL 87091 and LRG 41. However, cocoon formation was significantly lower (9.00–14.70%) when the *H. armigera* larvae were fed on ICPB 2042, ICPL 332WR and ICPW 125 as compared to that on ICPL 87119 (Table 1). Adult emergence of *C. chlorideae* was highest when the *H. armigera* larvae were reared on flowers/pods of ICP 7035 (40.33%), followed by LRG 41 (39.67%). Least adult emergence was recorded on ICPW 125 (3.33%), followed by ICPB 2042 (5.33%). Longevity of *C. chlorideae* adults was maximum when reared on *H. armigera* larvae fed on ICPL 87091 (29.70 days), followed by ICP 7035 (29.00 days). The adult longevity was significantly lower in *C. chlorideae* reared on *H. armigera* larvae fed on ICPW 125 (10.70 days) and ICPB 2042 (14.33 days) than those fed on ICP 7035 and ICPL 87091.

## Discussion

Effectiveness of natural enemies for controlling insect pests varies across crops, and different genotypes of the same crop (Sharma 1993; Sharma et al. 2003; Kaur et al. 2004; Dhillon and Sharma 2007). The present studies showed that the parasitization efficiency of the *H. armigera* larval parasitoid, *C. chlorideae* varies across different genotypes of pigeonpea. The host genotype not only influenced the parasitization of the host larvae, but also the survival and development of *C. chlorideae*, which may be because of the effect of the morphological and biochemical characteristics of the host genotype on *H. armigera* larvae, and the indirect effects on *C. chlorideae*. Percentage parasitization was greater under no-choice conditions as compared to that under multi-choice conditions, which may be largely because of non-availability of alternate host plant to the females of *C. chlorideae*. When the parasitoids were given a choice of all the test genotypes under multi-choice conditions, greater numbers of parasitoids were involved in seeking the host larvae, resulting in intraspecific competition, which might have resulted in lower levels of parasitism under multi-choice conditions. Competition is one of several factors influencing the effectiveness of parasitoids (Sirot and Bernstein 1996).

The *H. armigera* larvae feeding on susceptible genotypes, ICPL 87 and ICPL 97119 suffered significantly greater parasitization by *C. chlorideae* than the other genotypes tested under no-choice, dual-choice and multi-choice conditions, suggesting that host genotype plays a significant role in the effectiveness of *C. chlorideae* in pasitization of *H. armigera* larvae. Kaur et al. ( 2004) reported that as the incidence of a pest increases, the parasitoid activity also increases. In the present studies, the increased parasitization of *H. armigera* on the susceptible genotypes may also be influenced by survival of more numbers of *H. armigera* larvae on the susceptible genotype and lack of resistance to the herbivore, and possibly production of greater amounts of the volatile compounds that attract the *C. chlorideae* females for parasitization (War et al. 2011).

Clustering type of podding habit of ICPL 87 possibly provided more surface area for the movement of the *H. armigera* larvae, rendering them more prone to parasitization by *C. chlorideae*. ICPL 87119 - which has thicker pod walls, possibly hindered the entry of the *H. armigera* larvae into the pods making them more prone to parasitization by *C. chlorideae*. Creeping type growth habit and thick plant canopy of the *C. scarabaeoides* accession, ICPW 125 recorded lowest parasitization under no-choice, dual-choice and mulit-choice conditions, which may be due to the presence of long nonglandular hairs and low survival of *H. armigera* larvae because of high levels of resistance to *H. armigera* (Aruna et al. 2005; Sujana et al. 2008; Sharma et al. 2009), and a different blend of the chemicals on the pod surface (Green et al. 2002) and/or volatile compounds (Sharma et al. 2001) which influence the survival and development of the insect host, *H. armigera*. Behavior and parasitism rates of *Trichogramma pretiosum* (Riley) and *Trichogramma minutum* (Riley) in *H. armigera* are influenced by trichome and gossypol containing glands in cotton (El-Wakeil 2011), while t richomes in pigeonpea inhibit the searching behavior of *T. chilonis* Ishii (Romeis et al. 1998).

Pigeonpea genotypes also showed substantial effects on the survival and development of *C. chlorideae* parasitizing second-instar *H. armigera* larvae. Egg + larval period and pupal period were longest on the *C. scarabaeoides* accession, ICPW 125 and shortest postembryonic developmental period was recorded on the *H. armigera* susceptible genotype ICPL 87, suggesting that secondary metabolites ingested by the *H. armigera* larvae from the host genotype either directly affected the survival and development of the parasitoid, *C. chlorideae* or through suboptimal prey because of poor growth of the *H. armigera* larvae due the adverse effects of the secondary metabolites, and/or poor nutritional quality of the insect and plant host. Changes in biochemical composition of host plants in response to herbivory also influence the growth and survival of herbivores (Gange and Brown 1989; Whitaman et al. 1991), which in turn influences the activity and abundance of natural enemies (Bloem and Duffey 1990). Sithanantham et al. ( 1982) observed that parasitism of *H. armigera* larvae in chickpea was lower on the resistant genotypes than on the susceptible ones. The results of the present studies suggested that the ICPW 125, ICPB 2042 and ICPL 332WR are not compatible with the *H. armigera* larval parasitoid *C. chlorideae*.

Parasitization of *H. armigera* larvae by *C. chlorideae* was greater under no-choice, dual-choice and/or multi-choice conditions on ICPL 87, ICPL 87119 and ICPL 87091, which are susceptible to *H. armigera* than on the resistant genotypes ICPL 332WR, ICPL 84060, LRG 41 (Kumari et al. 2010a). Survival, development of the parasitoid was better on ICPL 87119, LRG 41, ICP 7035 and ICPL 87091 than on ICPL 332WR, ICPL 84060, ICPB 2042 and ICPW 125 - the pod borer resistant genotypes. The genotypes ICPL 87, ICPL 87119, LRG 42 and ICPL 87091, that are hospitable to *C. chloridae*, are more suitable for use in integrated pest management to minimize the damage by *H. armigera* in pigeonpea. These varieties have also been released for cultivation to the farmers and have acceptable grain quality, except ICPL 87091. In view of the variation observed in genotypic resistance to *H. armigera*, there is need for in-depth studies to assess the genotypic compatibility with the natural enemies of the crop pests for integrated pest management.

The larvae of *H. armigera*, which escape parasitization continue to develop and produce the next generation. The surviving population becomes the source for future generations of the parasitoids, which develop in the parasitized larvae and contribute to the natural balance between the pest and the natural enemies. The continuous and cumulative effect of the natural enemies on insect pests reduces the overdependence on synthetic pesticides for pest management, and thus, contributes to environment conservation. The genotypes that are compatible with the natural enemies may also be used by the organic farming community for producing food free from pesticides.
